# Comparison of the Hook Plate versus TightRope System in the Treatment of Acute Type III Acromioclavicular Dislocation

**DOI:** 10.1155/2022/8706638

**Published:** 2022-12-05

**Authors:** Abdulrahim Dündar, Deniz İpek

**Affiliations:** Hitit University Erol Olçok Training and Research Hospital, Department of Orthopedics and Traumatology, Çorum, Turkey

## Abstract

**Introduction:**

The objective of this study is to compare the effectiveness of the clavicular hook plate (HP) technique and the minimally invasive coracoclavicular (CC) fixation with a TightRope (MITR) procedure in treating acute unstable distal clavicle dislocation.

**Method:**

MITR (minimally invasive TightRope) group had 21 patients, and the open reduction and internal fixation (HP) group included 23 patients. Researchers compared MITR and HP (hook plate) outcomes for the treatment of acute type III AC joint dislocation in a retrospective analysis. The patients were followed up at 1 3, 6, and 12 months postoperatively. Complications were analyzed such as redislocation, fractures, implant-related complications, or subacromial erosion. For the clinical outcomes, the visual analog scale (VAS) (0: no pain, 10: worst possible pain), Constant-Murley score (CMS) (100: no pain, 0: maximum pain), the average satisfaction score with their current shoulder function (range: 0–10), and the University of California at Los Angeles Shoulder score (UCLA) (>27 good/excellent <27 fair/poor) were recorded at the last follow-up.

**Result:**

There were 21 sufferers in the MITR group, which comprises 19 males and 2 females and 23 individuals in the HP group (20 men and 3 women), with average ages of 43.9 and 39.2, respectively. Age, sex, laterality, and the interval between injury and surgery did not significantly differ between the two groups (0.357, 0.792, 0.432, and 0.55, respectively). No statistically significant difference was found between the groups in terms of the VAS score and CMS score at one year postoperatively. The mean CCD at the initial trauma and last follow-up was not significantly different between the MITR and HP groups (*p*=0.365, *p*=0.412 respectively).

**Conclusion:**

For treating acute type III AC dislocations, the minimally invasive TightRope (MITR) system and the hook plate technique were great options. However, the minimally invasive TightRope system showed further benefits such as reduced reoperation for implant removal and reduced risk of subacromial distal clavicle osteolysis.

## 1. Introduction

Dislocation of the acromioclavicular (AC) joint is a common injury, especially in physically active people, and it affects men five times more often than women. The most popular method of diagnosing AC joint dislocations is the Rockwood classification, which ranks acromioclavicular joint dislocations from type I to type VI based on the amount and direction of acromioclavicular joint injury or displacement of distal clavicle dislocation. Acromioclavicular joint dislocations (ACJs) of type III are further subdivided into type IIIA, or horizontally stable injuries, and type IIIB, or horizontally destabilizing injuries [1]. For type IIIA injuries, nonsurgical therapy is advised, whereas type IIIB injuries are recommended for surgical treatment [2]. The optimum treatment for acute AC dislocation is still debatable despite the high occurrence of the condition [3]. For the treatment of acromioclavicular dislocations, various surgical procedures, including the Weaver-Dunn method, coracoclavicular ligament fixation, auto-grafts, and improved graft patency (synthetic grafts), clavicular hook plate fixation, tension banding, fixation with the Endobutton, Kirschner wires fixation, and others techniques have all been suggested [4].

The two most popular techniques for treating AC dislocations are the hook plate (HP) operation and the TightRope (TR) surgery. Both methods produce outputs that are secure and efficient. The hook plate method has drawbacks too, including discomfort, functional restrictions, subacromial impingement syndrome, rotator cuff injuries, and a procedure to eradicate the plate through surgery [5]. There is currently no established gold standard for AC joint dislocations, even though all of the abovementioned treatments are important. However, there is debate about whether these procedures are clinically superior, and several problems have been described [6].

The purpose of this study was to evaluate the clinical and radiographic outcomes between TightRope system and open reduction and hook plate fixations for the treatment of acute Rockwood type III acromioclavicular joint dislocation.

The hypothesis of our study is open reduction and hook plate fixation, and the TightRope system yields comparable satisfactory radiographic and clinical results in the treatment of acute Rockwood type III AC joint dislocation. However, TightRope fixation not requires a second surgery for the removal.

## 2. Materials and Methods

**Figure 1:**
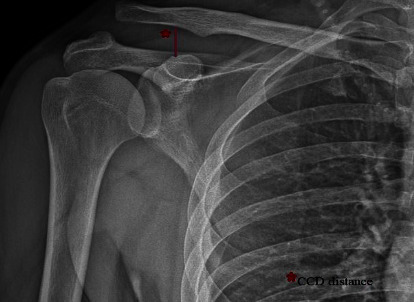
Measurement of the coracoclavicular distance (CCD) of a patient with Rockwood type III acromioclavicular joint dislocation on the preoperative anterior-posterior view. The CCD is the vertical distance between the coracoid's uppermost border and the inferior border of the clavicle.

In this retrospective study, 44 patients with Neer type III acromioclavicular joint dislocations were analyzed between January 2016 and March 2022 at our institute. MITR (minimally invasive TightRope) group had 21 patients, and the open reduction and internal fixation (HP) group included 23 patients. Researchers compared MITR and HP (hook plate) outcomes for the treatment of acute type III AC joint dislocation in a retrospective analysis. The following criteria were used by researchers to choose the patients:

 
**Inclusion criteria** are as follows: (1) Adult with acute, closed, and unilateral injuries; (2) fixation by minimally invasive TightRope or clavicular hook plating; (3) normal shoulder function before the dislocation; (4) no concomitant injuries; (5) age of 18 to 60 years; and (6) patients with regular follow-up more than 12 months at last postoperatively. Inclusion in this study was limited to 44 participants who met the criteria.
 
**Exclusion criteria** are as follows: (1) abnormal shoulder function before dislocation; (2) open injury; (3) 2 weeks after injury; (4) fracture of the other parts of the shoulder or limb; (5) follow up less than 12 months; (6) >3 weeks after injury; and (7) patients with the previous operation of the shoulder.
 
**Clinical evaluation**: The patients were followed up at the 1 3, 6, and 12 months postoperatively. Complications were analyzed such as redislocation, fractures, implant-related complications, or subacromial erosion. For the clinical outcomes, the visual analog scale (VAS) (0: no pain, 10: worst possible pain), Constant-Murley score (CMS) (100: no pain, 0: maximum pain), the average satisfaction score with their current shoulder function (range: 0–10), and the University of California at Los Angeles Shoulder score (UCLA) were recorded at the last follow-up.
 
**Radiographic evaluation:** To assess any remaining vertical AC joint instability, standard anterior and posterior radiographs of the surgical shoulder were taken at the last follow-up. Concerning the height of the acromion, the vertical displacement of the clavicle was measured (Figure 1). Researchers defined reduction, subluxation, and redislocation as no displacement, 50% displacement, and redislocation, respectively, concerning the height of the acromion.

### 2.1. HP Fixation Surgical Technique

**Figure 2:**
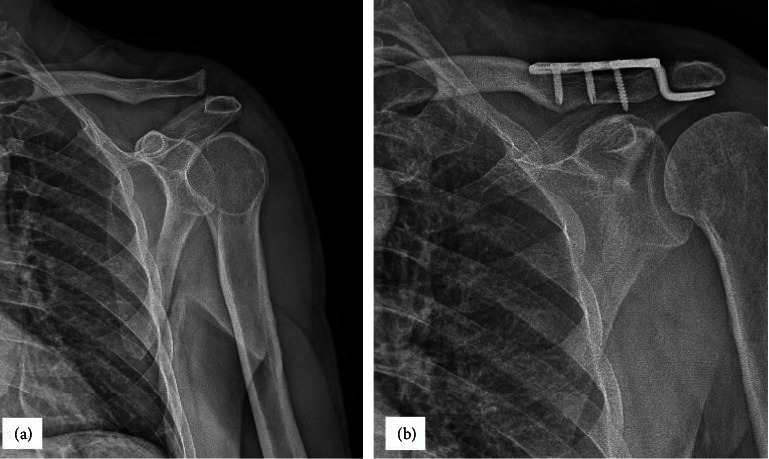
(a) The preoperative anterior and posterior view of a 32-year-old male patient with Rockwood type III acromioclavicular joint dislocation after a motorcycle accident injury. (b) 6-month postoperative plain radiograph showing good maintenance of joint reduction with hook plate fixation.

Under general anesthesia, the patients were positioned on a beach chair. The acromioclavicular joint dislocation was then visible after a transversal skin incision of 6 cm from the proximal clavicle to the acromion. Afterward, any articular cartilage fragments or hematomas were removed from the AC joint. Some Kocher forceps were used to temporarily secure the distal end of a plate to the clavicle after inserting the hook end of the plate underneath the acromion. The anterior shoulder reduction condition, depth of hook plate, and plate orientation were verified by fluoroscope with two aspects. Afterward, two or three locking screws were used to secure the hook plate fixation to the clavicle. To verify the plate placement, hook length, and screw length, as well as the reduction level, intraoperative fluoroscopy was performed (Figure 2).

### 2.2. MITR Surgical Technique

**Figure 3:**
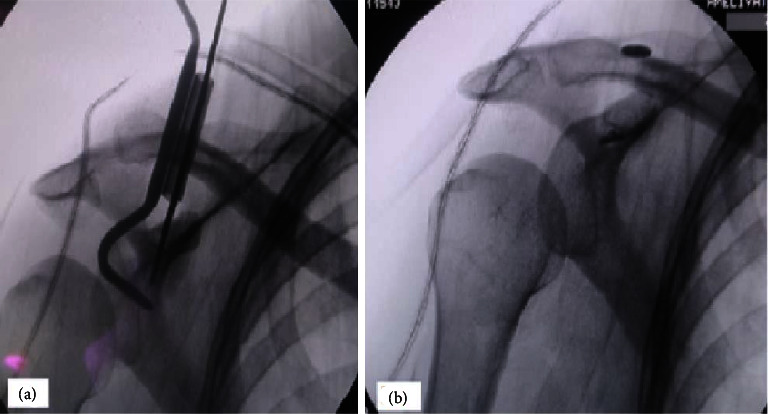
(a)-(b) Depicts the intraoperative procedure process.

Under general anesthesia and fluoroscopic guidance, the patient was positioned in a beach chair position. A 3 cm long vertical skin incision was made in line from the clavicle to the coracoid process. After AC joint reduction, a transclavicular-transcorocoidal guidewire was placed under fluoroscopy control. Then, the guidewire was drilled with a 4.0 mm hollow drill. The double bundle TightRope was inserted through bone tunnels with the help of a shuttle suture. The oblong button's location was confirmed under fluoroscopy control. The circular button was then lowered until it touched the upper clavicular surface. Lastly, sutures were used to fix the TR. Fluoroscopy was used intraoperatively to control the shrinkage (Figures 3(a) and 3(b)).

### 2.3. Postoperative Rehabilitation

After surgery, both groups were urged to perform gentle pendulum exercises for four weeks while wearing arm slings for safety and then progressing the operating range of the shoulder's movement.

The HP and MITR groups both underwent rehabilitation using the same techniques. After surgery, elbow, wrist, and hand exercises with a pendulum and full active mobility were permitted. Patients were permitted to move freely after the seventh week after having their arms could not move for a month using a sling. After 10 to 12 weeks, muscle-strengthening activities were initiated.

### 2.4. Statistical Analysis

SPSS software (Version 22, SPSS Inc., Chicago, IL, USA) was used for statistical analysis. Descriptive statistics were reported using numbers and percentages for categorical variables and mean ± standard deviation depending on data distribution for numerical variables. Mann-Whitney*U* test was used to determine the outcome between two independent groups since parametric test assumptions were not provided. Chi-square was used to compare the categorical variables. Differences between the two groups were considered significant at *p* < 0.05.

## 3. Results

There were 21 sufferers in the MITR group, which comprises 19 males and 2 females and 23 individuals in the HP group (20 men and 3 women), with average ages of 43.9 and 39.2, respectively. The patients' main demographic characteristics, time to surgery, and mean follow-up time are represented in Table 1. Age, sex, laterality, and the interval between injury and surgery did not significantly differ between the two groups (0.357, 0.792, 0.432, and 0.55, respectively). Mechanism of injury, CMS score, UCLA score (University of California at Los Angeles Shoulder score), VAS score, and initial and final CCD (Coracoclavicular distance) are represented in Table 2. With regard to the mean VAS scores at the final follow-up were 0.8 ± 1.2 and 1.2 ± 1.4 in groups MITR and HP, respectively(*p* = 0.442). The mean CMS (Constant-Murley scores) in groups MITR and HP was 90.5 ± 9.6 and 89.7 ± 2.7, respectively (*p* = 0.612) (Table 2). No statistically significant difference was found between the groups in terms of the VAS score and CMS score at one year postoperatively. The mean UCLA score in MITR and HP was 33.2 ± 1.5 and 32 ± 2.4, respectively (*p* = 0.734), and there was no statistically significant difference between the two groups. The average satisfaction score with their current shoulder function in MITR and HP was 8.2 ± 2.6 and 7.9 ± 1.4, respectively (*p* = 0.677), and there was no statistically significant difference between the two groups.

**Figure 4:**
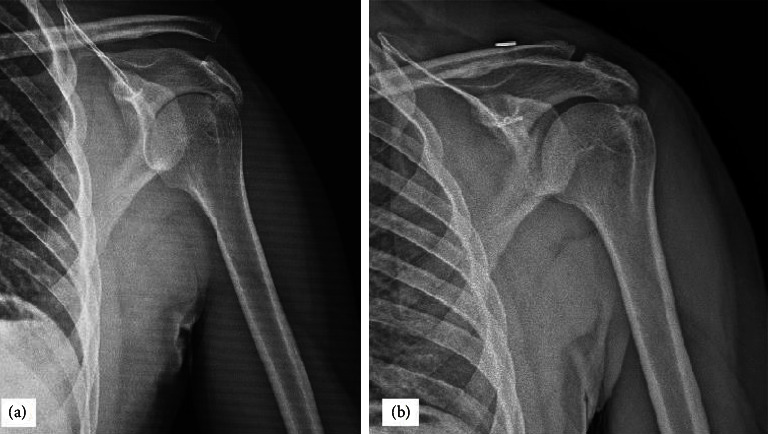
(a) The preoperative anterior and posterior view of a 42-year male patient, who had Rockwood type III acromioclavicular joint dislocation at his left shoulder due to the fall from standing height. (b) 6-month postoperative plain radiograph showing good maintenance of joint reduction with minimally invasive TightRope.

**Figure 5:**
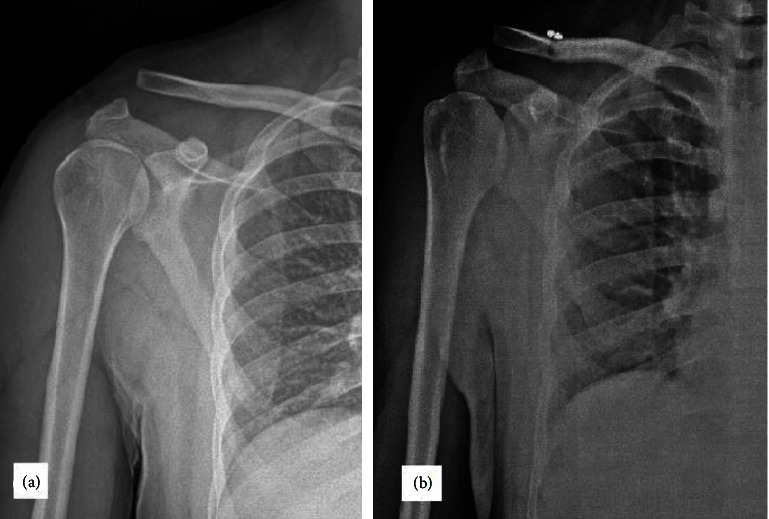
Serial radiographs of a 36-year-old man treated with a minimally invasive TightRope system. (a) Preoperative anterior-posterior radiograph showing type III AC dislocation. (b) 1-month postoperative follow-up with reduction failure.

CCD was measured consecutively and recorded. In the MITR group, mean CCD was reduced from 20.9 ± 50.9 preoperatively to 8.9 ± 9.2 at the last follow-up (Figure 4). In the HP group, mean CCD was reduced from 21.2 ± 59.2 preoperatively to 8.3 ± 6.7 postoperatively. The mean CCD at the initial trauma was not significantly different between the MITR and HP groups (*p*=0.365). In the HP group, 4 (17%) out of 14 patients had subacromial erosions and osteoarthritis of the acromioclavicular joint. 14 of the hook plate were removed after surgery at 4th and 5th months, and 9 patients declined to remove the hook plate. All of the reoperations in the HP group (14 out of 23) were due to implant removal, whereas the MITR groups have only one revision surgery, which was made for redislocation (Figure 5) (1/21).

In the current study, second surgery developed in 1 (4.7%) and 14 (60.8%) patients in the MITR and HP groups, respectively, and there was statistically significant difference between the two groups (*p* < 0.05).

The mean (CMS) Constant-Murley scores in groups MITR and HP were 90.2 ± 9.9 and 89.2 ± 3.5 (*p*=0.630), respectively. The CCD was measured consecutively and noted for statistical analysis. The mean CCD at the initial trauma was not significantly different between the MITR and HP groups (*p*=0.365). With regard to the mean CCD, no statistically significant difference was found between the MITR and HP groups at the final follow up (*p*=0.412).

## 4. Discussion

The key finding of the current study is that both the minimally invasive TightRope and the HP fixation may effectively be used for type III AC dislocations, with good to outstanding functional outcomes. The tension the HP technique put on the lower side of the acromion, and the second operation for implant removal, according to the researchers, may have contributed to a little higher predisposition to a VAS (visual analog scale) for the hook plate procedure (the mean VAS scores at the final follow-up were 0.8 ± 1.2 and 1.2 ± 1.4 in groups MITR and HP, respectively).

The hook plate method was widely used to treat acromioclavicular joint dislocation. Numerous HP fixation studies have reported good outcomes [7]. The HP method was used in future research to treat severe highest-degree acromioclavicular joint dislocations. Results were satisfactory following a 24 month (Constant-Murley Score CMS: 90.19 ± 7.79) [8]. Jensen et al. [9] treated 30 acute Rockwood type III acromioclavicular joint separations by using HP and had a good result (mean CMS of 92.4). In the current study, similar to the literature, the mean CMS of patients who underwent hook plate was found 89.7 ± 2.7 at the one year postoperatively.

According to the study by McConnell et al. [10], the HP method came the closest to returning the AC joint's biomechanics to normal. Although the results of the HP treatment have been satisfactory to exceptional, numerous studies have noted several problems, including flexor injury, redislocation, and degeneration of the acromioclavicular joint [11].

The clavicular hook plate had good outcomes, but it has the potential to injure the rotator cuff and create acromial dislocations and subacromial impingement. Additionally, due to the high probability of these problems with long-term fixation, a further procedure must be carried out to remove the hook plate [12]. Renger et al. [13] found that radiological analysis revealed acromial osteolysis in three patients (6.8%), and that during mobilization, 30 patients (68%) complained of pain, a scraping sensation, and a reduction in range of motion due to impingement. Following the removal of the hook plate, all implant-associated discomforts have vanished.

AC dislocations have been surgically treated with the TightRope approach and yielded satisfactory results [14]. However, the TR method's primary benefit is that the implant can be removed without requiring a second surgery. In certain studies, the TightRope technique performed better than the hook plate technique [15].

The MITR group in the current trial attained excellent to exceptional with favorable patient outcomes and an average CMS of 90.5 at the end of maintenance. Comparable results were reported by Jensen et al. [9].

Despite the TR technique's good postoperative outcomes, problems have also been noted, most likely due to a preliminary reduction, displacement of a button, or insufficient curing of fractured bones [16]. Redislocation is a frequent issue with the TR approach [17]. In the current investigation, the acromioclavicular joint was redislocated in one patient of minimally invasive TightRope due to the displacement of the coracoid buttons.

According to researchers, the two group's complication rate was lower than in the studies mentioned above, and there was no statistically significant difference in the complication between the two groups. From the researcher's perspective, the main issue with hook plate fixation was shoulder impingement (syndrome involving tendonitis) in motion. In order to prevent more acromial erosion, and enhance recovery of shoulder movement, subsequent surgical removal was performed on 14 of the research participants.

Although the HP fixation has a straightforward and uncomplicated approach, there are several worries that HP could cause shoulder discomfort and dysfunction. As a result of the HP, rotator cuff damage, subacromial osteolysis, osteoarthritis of the ACJ, and implant failure may occur, as well as impingement syndrome [18].

Similar to prior studies, nonrigid TightRope fixation's clinical outcomes were comparable to rigid fixation with HP in the current investigation. However, one of HP's primary worries is another operation required to remove the osteoarthritis of the ACJ and subacromial erosion [19].

There were several limitations, including the following: this study consisted of a small series and a relatively short follow-up duration. Second, this is a nonrandomized retrospective study design. The results of both methods may need to be confirmed in the future by a larger sample size and prospective controlled study.

## 5. Conclusion

For treating acute type III AC dislocations, the minimally invasive TightRope (MITR) system and the hook plate technique were great options. In the current investigation, the radiological and clinical outcomes of the MITR and HP procedures were comparable. Based on the findings of this study, it is possible to repair unstable distal clavicle dislocations using both surgical techniques of nonrigid TightRope fixation and AO clavicular hook plate with excellent functional outcomes. However, the minimally invasive TightRope system showed further benefits, such as reduced reoperation for implant removal and reduced risk of subacromial distal clavicle osteolysis. Long-termfollow-up is required to compare clinical and radiological outcomes between the two groups.

## Figures and Tables

**Table 1:** Main demographic characteristics.

Parameter	MITR group	HP group	*p* values
Number of patients	21	23	—
Age (years)	43.9 ± 11	39.2 ± 8.7	0.357^*a*^
Sex (M/F)	19/2	20/3	0.792^*b*^
Right/left	13/8	13/10	0.432^*a*^
From injury to surgery (days)	3.9 ± 3.1	4.1 ± 2.2	0.554^*a*^
Mean follow-up (months)	19.9 ± 3.1	20.1 ± 2.2	0.593^*a*^

Values are presented as number, mean (range), or number (%). ^*a*^Mann-Whitney *U* test, ^*b*^Chi-square test.

**Table 2:** Comparison of injury-related data, VAS and CMS score between MTRL and HP groups.

Parameter	*M* Group	*H* Group	*p* values
Number of patients	21	23	—
Redislocation (%)	1 (4.7)	0	—
Subacromial erosion	0	4 (17.3)	—
Implant removal	0	14 (60.8)	—
Second operation	1 (4.7)	14 (60.8)	<0.05^*b*^
Mechanism of injury (%)			0.56^*b*^
Fall	13 (61.9)	16 (69.5)	
Accident (bicycle, traffic, sport)	8 (38)	7 (30)	
Postoperative CMS score	90.5 ± 9.6	89.7 ± 2.7	0.612^*a*^
Postoperative VAS score	0.8 ± 1.2	1.2 ± 1.4	0.442^*a*^
Postoperative UCLA score	33.2 ± 1.5	32 ± 2.4	0.734^*a*^
Satisfaction shoulder score	8.2 ± 2.6	7.9 ± 1.4	0.677^*a*^
Initial CCD (mm)	20.9 ± 50.9	21.2 ± 59.2	0.365^*a*^
Final CCD (mm)	8.9 ± 9.2	8.3 ± 6.7	0.412^*a*^

Values are presented as number, mean (range), or number (%). CCD: coracoclavicular distance. CMS: Constant-Murley score. VAS: visual analog scale. UCLA: University of California at Los Angeles Shoulder score. ^*a*^Mann-Whitney*U* test, ^*b*^Chi-square test.

## Data Availability

The data used to support the findings of this study are restricted by the ETHICS BOARD NAME in order to protect PATIENT PRIVACY or ENDANGERED SPECIES. Data are available from Abdulrahimdündardundarabd@hotmail.com for researchers who meet the criteria for access to confidential data.
